# Deamidation Reactions of Asparagine- and Glutamine-Containing Dipeptides Investigated by Ion Spectroscopy

**DOI:** 10.1007/s13361-016-1462-5

**Published:** 2016-09-13

**Authors:** Lisanne J. M. Kempkes, Jonathan Martens, Josipa Grzetic, Giel Berden, Jos Oomens

**Affiliations:** 1 FELIX Laboratory, Institute for Molecules and Materials, Radboud University, Toernooiveld 7c, 6525 ED Nijmegen, The Netherlands; 2 Van ‘t Hoff Institute for Molecular Sciences, University of Amsterdam, Science Park 904, 1098 XH Amsterdam, The Netherlands

**Keywords:** Asparagine, Glutamine, Deamidation, Reaction mechanism, Infrared ion spectroscopy

## Abstract

Deamidation is a major fragmentation channel upon activation by collision induced dissociation (CID) for protonated peptides containing glutamine (Gln) and asparagine (Asn) residues. Here, we investigate these NH_3_-loss reactions for four Asn- and Gln-containing protonated peptides in terms of the resulting product ion structures using infrared ion spectroscopy with the free electron laser FELIX. The influence of the side chain length (Asn versus Gln) and of the amino acid sequence on the deamidation reaction has been examined. Molecular structures for the product ions are determined by comparison of experimental IR spectra with spectra predicted by density functional theory (DFT). The reaction mechanisms identified for the four dipeptides AlaAsn, AsnAla, AlaGln, and GlnAla are not the same. For all four dipeptides, primary deamidation takes place from the amide side chain (and not from the N-terminus) and, in most cases, resembles the mechanisms previously identified for the protonated amino acids asparagine and glutamine. Secondary fragmentation reactions of the deamidation products have also been characterized and provide further insight in – and confirmation of – the identified mechanisms. Overall, this study provides a comprehensive molecular structure map of the deamidation chemistry of this series of dipeptides.

**Electronic supplementary material:**

The online version of this article (doi:10.1007/s13361-016-1462-5) contains supplementary material, which is available to authorized users.

**Graphical Abstract:**
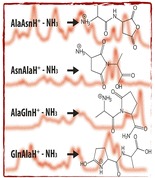
ᅟ

## Introduction

**Scheme 1:**
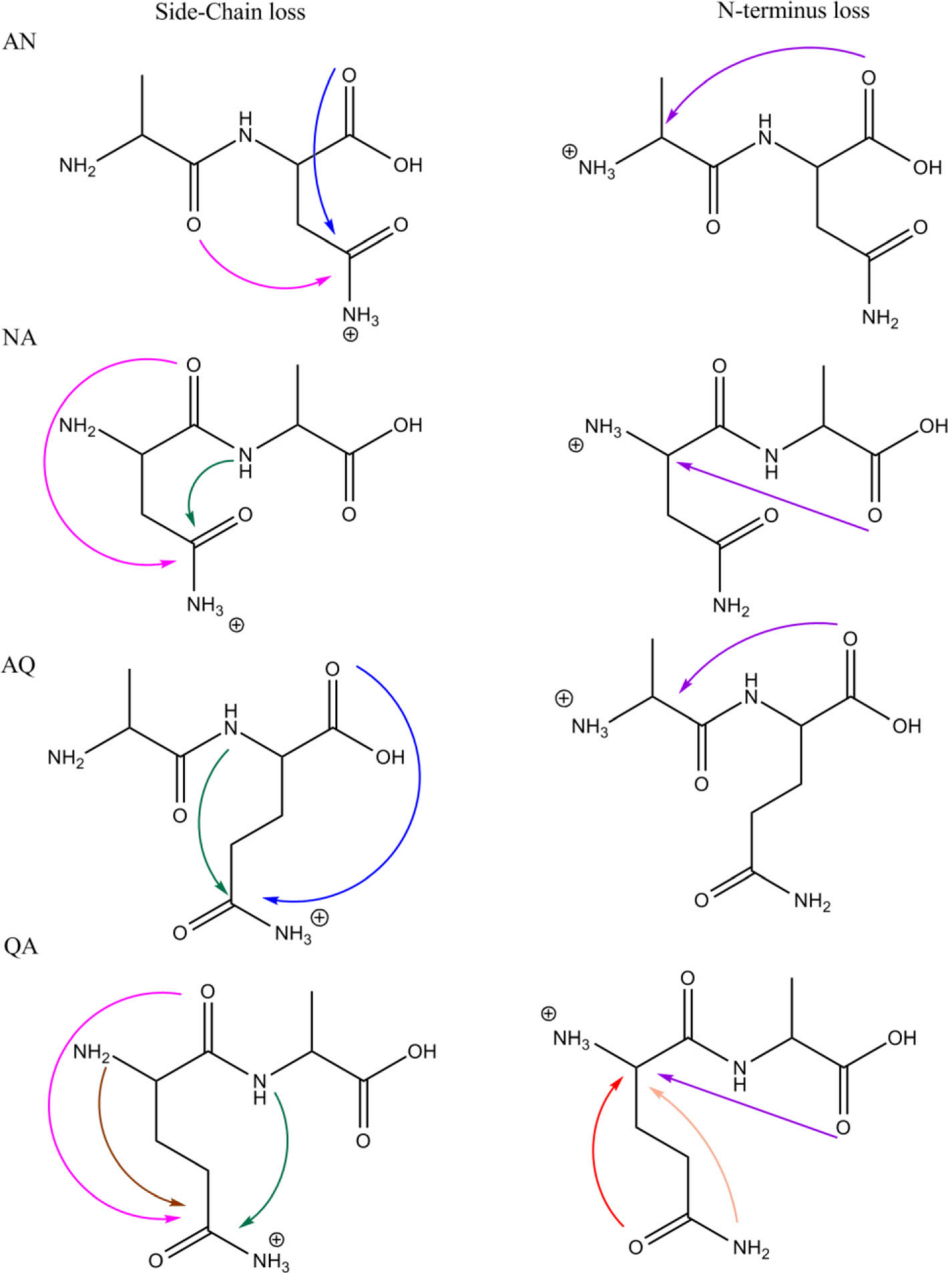
Possible reaction mechanisms for NH_3_ loss from the protonated dipeptides AlaAsn (AN), AsnAla (NA), AlaGln (AQ), and GlnAla (QA). Both ammonia loss from the side chain (left column) as well as from the N-terminus (right column) are taken into account. This Scheme only considers NH_3_ loss induced by nucleophilic attack leading to 5- or 6-membered ring products. Mechanisms leading to energetically less favorable 4-,7-, or higher membered ring structures have been considered but are not shown here. Analogous reactions are indicated by arrows of the same color. The dark and light brown arrows for QA indicate two different reactions leading to the same product

Under natural conditions, deamidation of glutamine (Gln) and asparagine (Asn) residues in proteins is a primary route for post-translational modifications, playing a role in several diseases, and is believed to relate to aging effects acting as a molecular clock [1–5]. Studies of the mechanistics of Asn and Gln deamidation in condensed media have shown that the reaction proceeds via cyclic imide intermediates (i.e., glutarimide for Gln and succinimide for Asn [3–5]. These imides are formed via nucleophilic attack by the adjacent peptidyl amide nitrogen onto the side chain carbonyl carbon atom with concomitant elimination of NH_3_ (green arrows in Scheme 1). Alternatively, for peptides with Gln as the N-terminal residue, partial deamidation in solution occurs by conversion of the Gln residue into a cyclic pyroglutamyl residue (2-pyrrolidinone-5-carboxylic acid) [6–8]. This type of reaction is indicated for GlnAla (QA) with the light and dark brown arrows in Scheme 1.

Also in the gas phase (i.e., as protonated ions in a mass spectrometer) Gln- and Asn-containing peptides are subject to facile NH_3_ loss under activation by collision induced dissociation (CID). In fact, loss of small neutral molecules including, in particular, NH_3_ and H_2_O commonly competes with backbone cleavage under slow heating conditions employed in MS-based peptide sequencing [9]. To better understand the mechanistics involved in deamidation of protonated peptides containing Gln and Asn, we employ here infrared ion spectroscopy to resolve the structures of the product ions resulting from NH_3_-loss from the protonated dipeptides of AlaAsn (AN), AsnAla (NA), AlaGln (AQ), and GlnAla (QA). The sensitivity and extensive MS^n^ capabilities of the ion trap mass spectrometer used in this study enable not only the characterization of MS/MS reaction products, but also of products resulting from further stages of CID (MS^n^). We thus construct a deep molecular structure map of the reaction network, where structural identification of product ions at different MS^n^ stages provides further consistency for the entire network.

In recent years, ion spectroscopy has emerged as a useful probe of ion structures of small gas-phase species and their reaction products after CID, being capable of distinguishing between isomers, protomers, and even conformers [10–23]. It has, for instance, been recently shown that b_2_-sequence ions from specific peptides containing Gln or Asn do not form along the common oxazolone dissociation pathway, nor via the alternative diketopiperazine pathway [24]. Although yet other alternatives had been suggested earlier [3, 25–27], cyclic imide structures formed through nucleophilic attack by the amide nitrogen atom of the Gln and Asn side chains was firmly established only after analysis of IR spectra of the product ions.

The mobile proton model for peptide dissociation proposes that the added proton, initially being localized on the most basic site, migrates upon mild collisional activation to a less favored protonation site [28–32]. For this reason, the deamidation reaction for the investigated dipeptides can conceptually take place from either the N-terminus or the side chain amide. For the protonated amino acids of Gln and Asn, it was recently established that NH_3_-loss occurs in both cases from the amide side chain and that deamidation is assisted by a nucleophilic attack leading to cyclization [10, 33]. Nonetheless, the reaction mechanism is not the same for the two amino acids, as the attacking nucleophile is the C-terminal carbonyl O-atom for Asn and the N-terminal N-atom for Gln. Following these reaction pathways, a 5-membered ring product ion is formed for both amino acids: 3-amino-succinic anhydride is formed from protonated Asn and pyroglutamic acid (2-pyrrolidone-5-carboxylic acid) is formed from protonated Gln.

Scheme 1 outlines for each of the four dipeptides studied here the possibilities for the formation of 5- and 6-membered ring structures after NH_3_-loss from either the amide side chain or the N-terminus. Attacks leading to 4-, 7-, or 8-membered ring structures were found to be energetically disfavored and to provide no agreement with the experimental IR spectra; in the interest of conciseness, we will not consider those structures further in the text of this report, although they were considered during the analysis of the spectra. As can be concluded from Scheme 1, for each of the dipeptides, three or more isomeric [M + H – NH_3_]^+^ structures are conceivable and, moreover, for each of them multiple protomers and conformers are generally possible, illustrating in general the complexity of the problem.

For protonated AN and AQ, the blue arrow in Scheme 1 indicates a deamidation mechanism analogous to that established for the protonated Asn amino acid [10, 33]. Similarly for protonated QA, nucleophilic attack of the N-terminal nitrogen onto the side-chain amide carbon (dark brown arrow) is analogous to the mechanism observed for the Gln amino acid [10]. Note that nucleophilic attack by the side chain nitrogen onto the α-carbon of the N-terminal residue (light brown arrow) leads to the same product and would thus be indistinguishable by spectroscopic characterization of the fragment ion; a similar ambiguity was encountered for the Gln amino acid. For NA, some of the mechanisms analogous to those indicated for QA would result in 4-membered ring species and are not included in Scheme 1.

Notable mechanistic studies, both experimental and theoretical, on the deamidation reactions occurring in the gas phase upon CID of protonated peptides include those reported in [7, 8, 34–36]. We note that these reactions have often been considered to be analogous to dehydration reactions from Glu- and Asp-containing peptides [34, 37]. A relatively large number of studies have addressed NH_3_-loss from peptides with an N-terminal Gln residue [6–8, 34], where the consensus is that the deamidation of the Gln side chain leads to the formation of a pyroglutamyl moiety following the brown arrow in the bottom left panel of Scheme 1. Deamidation of protonated AlaGln (AQ) is discussed by Harrison [34] and considered analogous to dehydration of AlaGlu (AE), although the structure of the resulting fragments could not be determined with certainty. The fragmentation chemistry of protonated ValAsn (VN) and ValGln (VQ) have been investigated by theoretical modeling at the density functional theory level [35]. Deamidation reaction mechanisms were predicted to be analogous for the two peptides, occurring from the side chain by a nucleophilic attack of the backbone amide oxygen onto the side chain carbonyl carbon. In Scheme 1, this mechanism is indicated for AN by the pink arrow; the reactions lead to 6- and 7-membered lactone ring structures for VN and VQ, respectively. Our analysis of deamidation of protonated AQ and AN presented below suggests a different mechanism. Armentrout and coworkers very recently reported a detailed investigation of the potential energy surface for NH_3_-loss from protonated AsnGly [36], establishing reaction pathways similar to those found here for [AsnAla + H]^+^.

## Experimental and Computational Methods

### IRMPD Spectroscopy

The experiments were performed in a modified 3D quadrupole ion trap mass spectrometer (Bruker, AmaZon Speed ETD, Bremen, Germany) coupled to one of the beam ports of the Free Electron Laser for Infrared eXperiments (FELIX) [38–40]. Protonated peptides are generated by electrospray ionization (ESI) and fragments are generated by collision induced dissociation (CID) and mass-selected using the standard MS^n^ routines of the instrument.

Peptide samples were purchased from GeneCust (Luxemburg) and used without further purification. For each experiment, a freshly prepared solution was used to avoid naturally occurring degradation of the dipeptides in the solution. Protonated dipeptide ions were generated using ESI from 10^–5^ – 10^-6^ M solutions in 50:50 acetonitrile:water and ~0.1% formic acid. Deamidated ions were obtained by subjecting the precursor ions to CID conditions for 40 ms using an amplitude parameter of approximately 0.3–0.5 V. Sequential fragmentation is induced by subjecting the mass-isolated deamidated [M + H – NH_3_]^+^ ions to a second stage of CID (MS^n^).

To record infrared spectra of the deamidated dipeptides and further fragmented product ions, Infrared multiple photon dissociation (IRMPD) spectroscopy [41, 42] has been applied after mass-isolation of the product ion of interest using the IR radiation from FELIX. Infrared absorption by the system causes an increase in the internal energy, which leads to frequency-dependent unimolecular dissociation. The dissociation yield at each IR wavelength is determined from three averaged mass spectra. An infrared vibrational spectrum is generated by relating the precursor and IR induced fragment ion intensities according to

$$ Yield={\displaystyle \sum {I}_{fragment}}/\left({\displaystyle \sum {I}_{fragment}+{I}_{precursor}}\right) $$

where the sum is over all fragments produced by IR irradiation. An assumed linear correction is applied for the frequency-dependent variations in the laser power. Tunable infrared radiation from FELIX is delivered in pulses of approximately 6 μs duration, 20–60 mJ energy, and at a bandwidth of ~0.5% of the center frequency at a repetition rate of 10 Hz. The frequency is calibrated using a grating spectrometer.

The IRMPD spectrum of protonated AQ – 17 is measured using the combination of FELIX with a Fourier transform ion cyclotron resonance mass spectrometer (FTICR-MS) [43, 44]. The experimental procedure is similar to that described above and has been detailed elsewhere [12, 21].

### Computational Chemistry

IR spectra are predicted and the molecular geometries are optimized for isomeric structures that were deemed possible by chemical intuition, including all likely protomers. Calculations on these first guess structures were performed at the density functional theory (DFT) level with the B3LYP/6-31++G(d,p) basis set, using Gaussian 09 revision D01 [45].

For the lowest energy structures of these [M + H *–* NH_3_]^+^ fragments, potential energy surfaces are further explored to identify the lowest energy conformers using a molecular mechanics/molecular dynamics (MM/MD) approach employing AMBER 12 [46]. After minimization within AMBER, a simulated annealing procedure up to 500 K was used. Five hundred structures were obtained in this procedure and grouped based on structural similarity using appropriate rms criteria to give 20–30 candidate structures. Next, these structures were each optimized at the B3LYP/6-31++G(d,p) level as described above. A more detailed description of the procedure can be found elsewhere [39]. For all optimizations on all geometries, the calculations were completed with electronic energy calculations at the MP2(full)/6-311 + G(2d,2p)//B3LYP/6-31++G(d,p) level at 298 K.

For the purpose of comparison with experimental spectra, the DFT computed harmonic vibrational frequencies were scaled by 0.975 and convoluted with a 20 cm^–1^ full-width-at-half-maximum Gaussian line shape.

## Results

### Alanine-Asparagine (AN)

As depicted in Scheme 1, three nucleophilic attacks are considered most likely to occur in the deamidation reaction of protonated AN. The C-terminal carbonyl oxygen can attack the asparagine side chain, leading to the 5-membered succinic anhydride structure 1 in Table 1. Alternatively, the oxygen from the amide linkage can attack the asparagine side chain, leading to a 6-membered ring structure, 2, which has been proposed on the basis of a computational study for the deamidation of protonated ValAsn [35]. A third possibility involves loss of NH_3_ from the N-terminus, resulting from an attack by the C-terminal carbonyl oxygen on the N-terminus, leading to the morpholine ring structure 3.

**Table 1:**
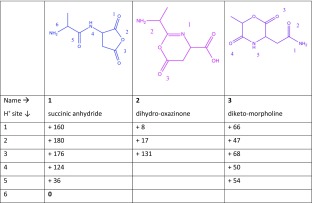
Deamidation of Protonated AlaAsn (AN).

Calculated relative free energies at the MP2(full)/6-311 + G(2d,2p)//B3LYP/6-31++G(d,p) level at 298 K for the different possible isomeric structures after NH_3_ loss, for each of which different protonation sites have been considered. The colors of the structures correspond with the colors of the arrows in Scheme 1, for example, the nucleophilic attack of the C-terminal oxygen on the side chain is indicated with a blue arrow, leading to structure 1 presented in blue in this table

For each of the resulting structures, multiple protonation sites are conceivable. The most likely ones are indicated with numbers in Table 1 and their relative computed energies are given. The 5-membered succinic anhydride structure, 1.6, protonated at the N-terminus is the overall lowest energy structure.

**Figure 1:**
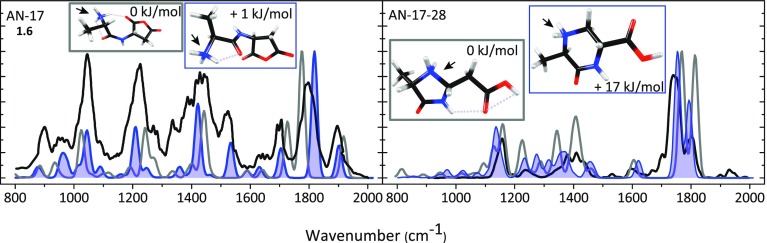
The experimental IRMPD spectra (*black*) of AN-17 (*left panel*) and AN-17-28 (*right panel*) compared with the best matching (*blue*) and the lowest energy (*grey*) calculated spectra. Of the two nearly iso-energetic 1.6 conformers in the left panel, we suspect the structure in blue to have the highest contribution to the experimental IRMPD spectrum (indicated by the *shading*). The protonation sites are indicated with an *arrow*

The left panel of Fig. 1 shows the experimental IRMPD spectrum (black) of the *m/z* 187 deamidation product ion (AN – 17) compared with the computed spectra of 1.6 in two nearly iso-energetic conformations (grey and blue). Supporting Information Figure S1 shows the experimental spectrum compared with computed spectra for each of the suggested structures in Table 1. Based on spectral matching, we propose to assign the anhydride structure (1.6) in blue as the structure of the ion present in the experiment (spectra of assigned structures are shaded throughout the paper). Note that this structural assignment is at odds with the dissociation mechanism proposed for ValAsn [35], which would lead to the oxazinone structure 2.1; clearly, the computed spectrum for this structure does not match the experimental one (see Figure S1). The difference between the spectra of the two conformers of 1.6 is most prominent for the band near 1530 cm^–1^, which is due to the N-H bending normal mode of the amide bond. For the identified structure shown in blue, the bands around 1820 and 1900 cm^–1^ are assigned as the symmetric and antisymmetric combination of C=O stretches of the succinic anhydride ring; the band around 1700 cm^–1^ is due to C=O stretching of the amide carbonyl.

Isolation and an additional stage of CID on the AN – 17 ion generates mainly a fragment at *m/z* 159, corresponding to AN – 17 – 28. We use IRMPD spectroscopy on this MS^3^ fragment ion in order to establish its structure, which can also provide further confirmation of the structure identified for AN – 17 above. The loss of 28 mass units can correspond to neutral loss of CO or C_2_H_4_. While the resolution of the mass spectrometer is not sufficient to distinguish between them, inspection of the proposed structure 1.6 for AN – 17 suggests that loss of CO is more plausible than loss of C_2_H_4_. The right panel of Fig. 1 shows the experimental IRMPD spectrum of AN – 17 – 28 together with calculated spectra for two low energy isomers (grey and blue) resulting from CO loss from 1.6. The blue-framed structure is the product of a nucleophilic attack by the N-terminal nitrogen onto the CH_2-_group of the succinic anhydride ring structure, leading to loss of CO from the ring and rearrangement to a 6-membered ring structure. The grey-framed structure is the result of a nucleophilic attack by the N-terminal nitrogen onto the CH in the succinic anhydride ring, leading to a 5-membered ring structure.

For the AN – 17 – 28 spectrum in grey, the vibration calculated at 1810 cm^–1^ is due to stretching of the carbonyl group in the ring, whereas that at 1760 cm^–1^ is due to the carboxyl C=O stretch. The weak band around 1600 cm^–1^ is due to NH_2_ bending. For the alternative AN – 17 – 28 structure (blue), the band near 1800 cm^–1^ is due to the carboxyl C=O stretch, whereas that at 1760 cm^–1^ is identified as C=O stretching of the carbonyl in the 6-membered ring. The peak around 1620 cm^–1^ is due to NH_2_-bending of the protonated secondary nitrogen. Although the experimental spectrum appears to match slightly better with the computed spectrum for the higher energy 6-membered ring structure, the similarity of the two calculated spectra does not truly allow us to identify one or the other and the spectrum may also be due to a mixture of both structures. Nonetheless, both isomers can conceivably be formed from the AN – 17 structure identified above, so that the analysis of the IR spectrum for AN – 17 – 28 provides additional support for the structural assignment of 1.6 as the AN – 17 fragment.

**Scheme 2:**
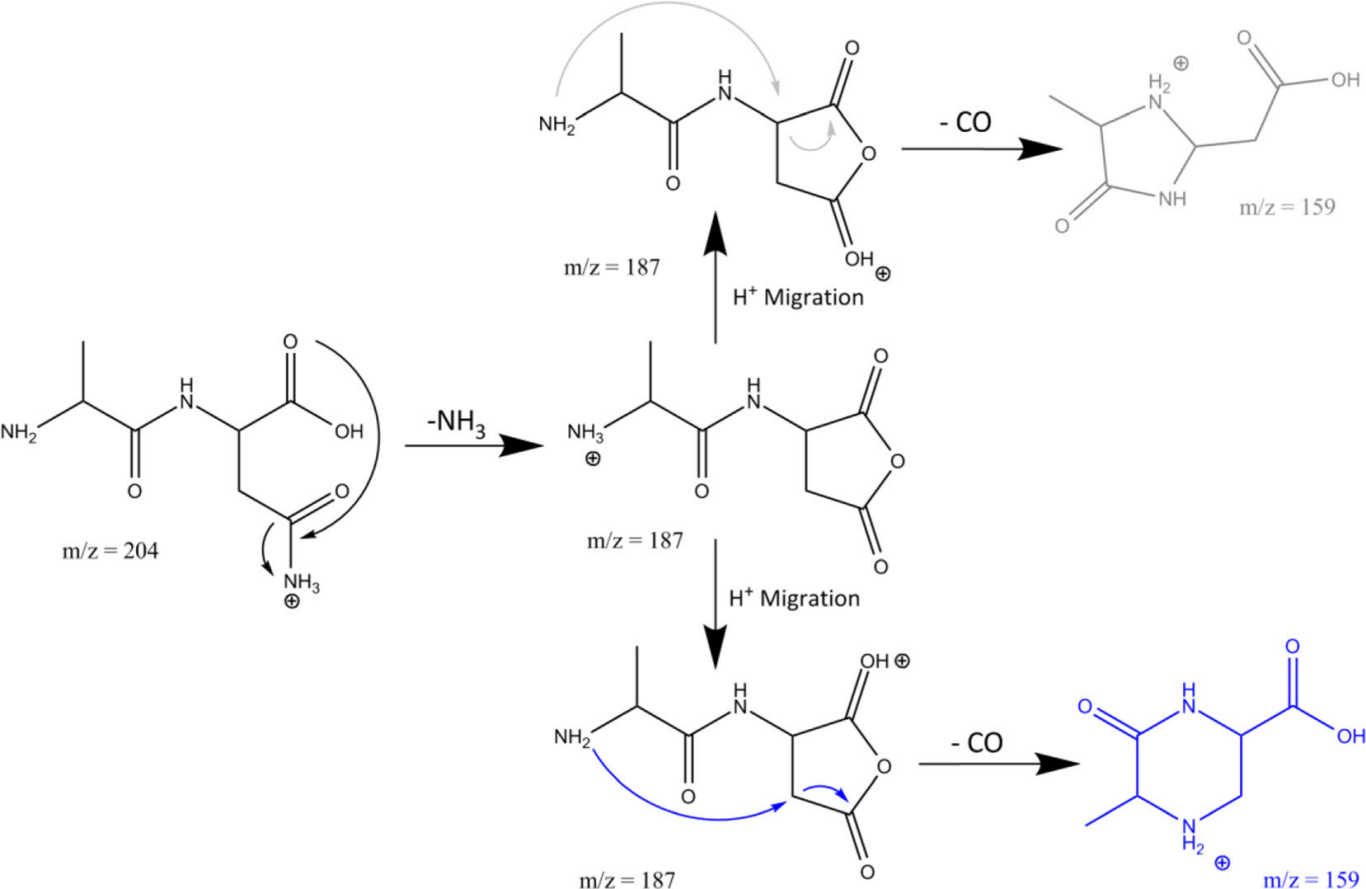
The suggested reaction mechanism for the fragmentation of protonated AN as suggested from the product ion structures identified from their IRMPD spectra

Scheme 2 shows the reaction mechanisms for the formation of AN – 17 and AN – 17 – 28, including pathways leading to both suggested isomers for the secondary neutral loss of 28 mass units.

### Asparagine-Alanine (NA)

Scheme 1 presents three possible reaction mechanisms for the deamidation of protonated NA. The amide linkage oxygen can attack the side chain leading to a 5-membered lactone ring, 4 in Table 2, the nitrogen in the amide linkage can attack the side chain leading to a succinimide ring, 5, or the C-terminal oxygen can attack the N-terminal α-carbon leading to 6. For each isomer, multiple protonation sites are again conceivable and the most prominent ones are shown with their relative computed energies in Table 2. Structure 5.1 represents the lowest-energy structure.

**Table 2:**
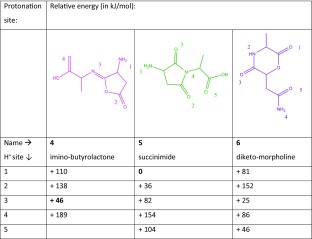
Deamidation of Protonated AsnAla (NA).

Calculated relative free energies at 298 K for different isomeric structures and protonation sites after loss of NH_3_. Colors of the suggested structures correspond to colors of the arrows showing the reaction mechanism in Scheme 1

**Figure 2:**
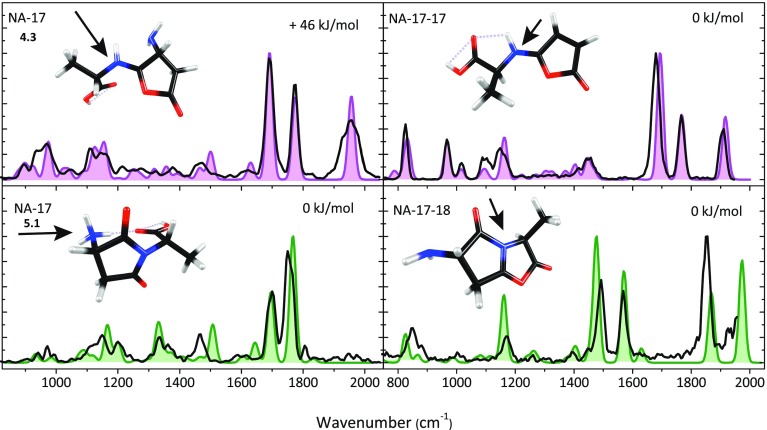
Experimental IRMPD spectra of NA – 17 (*left panel*), NA – 17 – 17 (*top right panel*) and NA – 17 – 18 (*bottom right panel*) compared with the best matching calculations. For NA – 17, two isomers are identified (*top panel and lower panel*) by separate analysis of the IR induced dissociation from NA – 17 into mass channels *m/z* 169 (H_2_O loss) and *m/z* 170 (NH_3_ loss) and are assigned to structures 4.3 (calculated spectrum in pink) and 5.1 (*green*). IRMPD spectra of the two product ions NA – 17 – 17 and NA – 17 – 18 have also been recorded (b*lack traces in the panels on the right*). The protonation sites are indicated with an *arrow*

Upon IRMPD of protonated NA – 17, fragments are produced at *m/z* 170 (NA – 17 – 17) and at *m/z* 169 (NA – 17 – 18), and it was noticed that the spectral responses in each of these channels are significantly different. This is suggestive of there being two different isomers of the precursor NA – 17 ion, each having a different IR spectrum and undergoing different fragmentation (i.e., neutral loss of either NH_3_ or H_2_O). The top left panel of Fig. 2 shows the experimental spectrum of NA – 17 as derived from the mass channel corresponding to sequential loss of 17, the bottom left panel shows the IRMPD spectrum derived from sequential loss of 18. The two spectra are analyzed by comparison with computed spectra for the series of isomers and protomers listed in Table 2. The best match for the spectrum observed in mass channel 170 is provided by the computed spectrum of lactone structure 4.3 (pink), whereas the spectrum observed in mass channel 169 is best reproduced by the computed spectrum for succinimide structure 5.1 (green). These structures match those recently established for deamidation of protonated AsnGly on the basis of TCID measurements and extensive theoretical modeling [36]. No match was found with spectra computed for the morpholine ring structures 6, as further shown for its lowest energy protomer 6.3 in Supporting Information Figure S2.

The most striking difference between the two individual experimental spectra for protonated NA – 17 is the band at 1955 cm^–1^, which is assigned to the stretching of the carbonyl group in the lactone ring structure of 4.3. More subtle differences are observed for the doublet of bands in the 1600–1800 cm^–1^ range; the computed spectra for the lactone 4.3 and succinimide 5.1 structures closely reproduce these differences. The most prominent deviation between experiment and theory is the ~50 cm^–1^ shift encountered for the band near 1500 cm^–1^ in the spectrum of the succinimide structure (green), which is due to the NH_3_ bending mode. The computed spectrum of 4.3 (pink) indicates that the band around 1775 cm^–1^ is due to C=O stretching of the carboxyl group, the band at 1690 cm^–1^ corresponds to C=N stretching of the imine bond and the weaker band near 1630 cm^–1^ is due to NH_2_ bending of the amine. For 5.1, the computations indicate that the band around 1766 cm^–1^ is the antisymmetric combination of succinimide C=O stretches, the band at 1695 cm^–1^ is due to the C=O stretch of the carboxyl group, and the bands at 1644 cm^–1^, 1604 cm^–1^, and 1507 cm^–1^ are due to bending modes of the NH_3_ group.

**Scheme 3:**
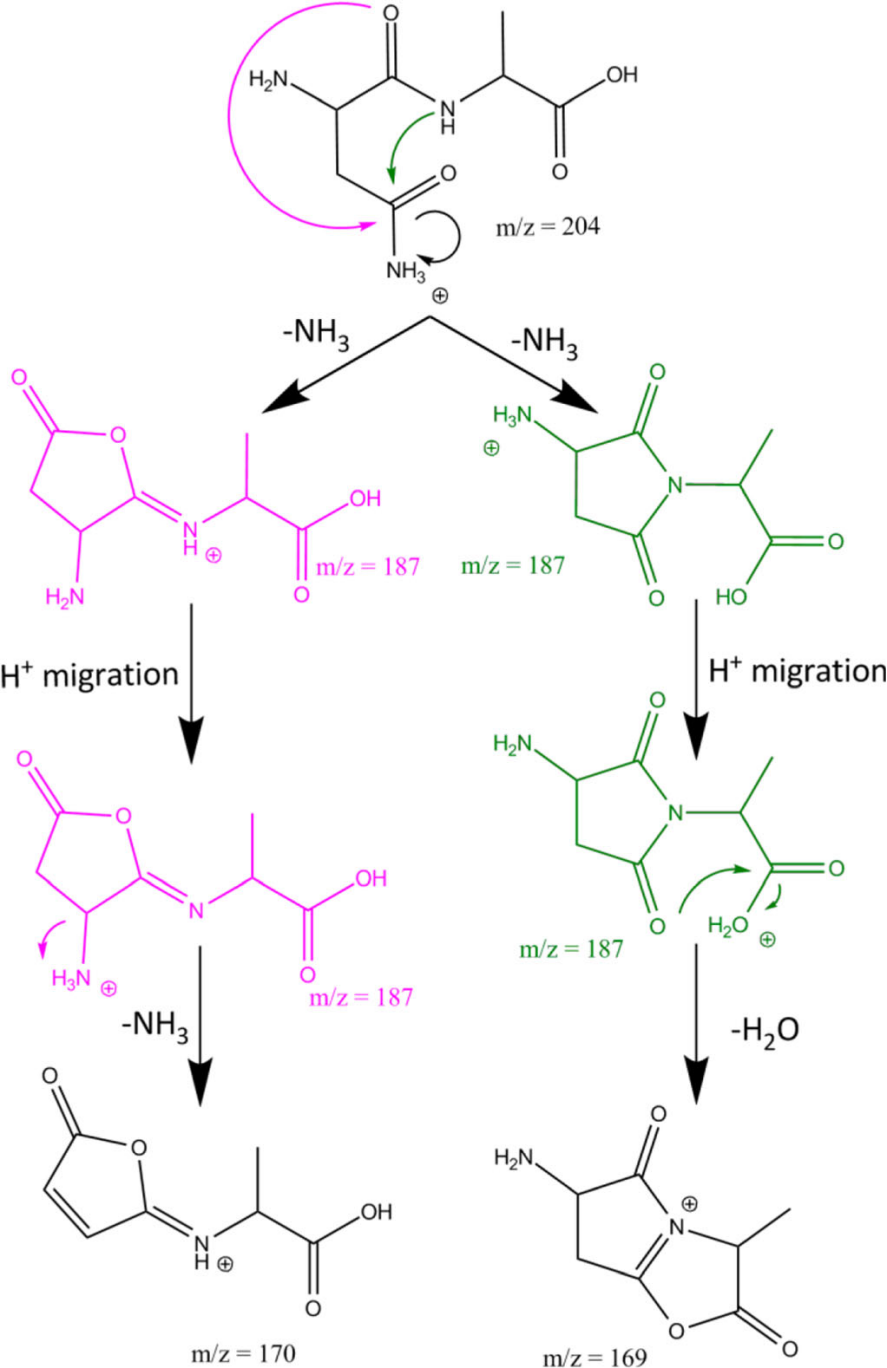
Suggested reaction mechanism for the fragmentation of protonated NA as derived from the product ion structures identified from their IRMPD spectra. For NA – 17, two isomers are found, each exhibiting different secondary breakdown reactions

IRMPD spectroscopy was then applied to the MS^3^ fragments NA – 17 – 17 and NA – 17 – 18 to further follow the dissociation cascade and to support the structural assignment for the two isomers of NA – 17 above. The spectrum of the NA – 17 – 17 (*m/z* 170) MS^3^ fragment is shown in the top right panel of Fig. 2 and matches excellently with the spectrum predicted for an imine substituted furanone structure, with protonation on the imine nitrogen (see Scheme 3). This suggests that 4.3 undergoes further fragmentation by loss of another ammonia molecule by direct cleavage of the amine substituent on the lactone ring. In contrast, structure 5.1 undergoes subsequent loss of H_2_O. The IRMPD spectrum of the NA – 17 – 18 (*m/z* 169) fragment is well reproduced by the spectrum predicted for a double 5-membered ring structure shown in the lower right panel of Fig. 2. The identification of this fragment structure suggests that water is detached from the carboxylic acid group with concomitant nucleophilic attack of one of the succinimide carbonyl oxygen atoms onto the carboxylic carbon (see Scheme 3). Recording the spectra of the MS^2^ and MS^3^ fragments thus yields a complete and consistent picture of the deamidation reactions, as summarized in Scheme 3.

For NA – 17 – 17, the band around 1915 cm^–1^ is assigned to C=O stretching in the carbonyl group and the band around 1470 cm^–1^ is assigned to C-H bending in the methyl group of the molecule. For the calculated NA – 17 – 18 spectrum, the peak around 1973 cm^–1^ is assigned to C=O stretching in the oxazolone ring, whereas the peak at 1868 cm^–1^ is assigned to the C=O stretch of the pyrolidone ring. The peak at 1629 cm^–1^ is assigned to NH_2_ bending, the peak at 1570 cm^–1^ is assigned to C=N stretching, the peak at 1477 cm^–1^ is assigned to C-O stretching in the lactone ring, and the peak at 1160 cm^–1^ is due to C-H bending.

The identification of the various structures leads to a suggested reaction mechanism as shown in Scheme 3. For the pathway depicted in pink, the oxygen at the amide linkage attacks the asparagine side chain, inducing the detachment of the first ammonia molecule. This leads to the imino-butyrolactone structure 4 analogous to the deamidation reaction of the amino acid asparagine [10]. The second ammonia molecule is lost by cleavage of the amine substituent on the lactone ring after transfer of the proton from the imine nitrogen. For the parallel reaction depicted in green in Scheme 3, the first step is a nucleophilic attack of the amide bond nitrogen on the asparagine side chain, leading to the succinimide structure 5, which was also observed in the deamidation reaction of asparagine-containing peptides in solution [3–5]. Subsequent fragmentation involves the loss of a water molecule from the C-terminus.

### Alanine-Glutamine (AQ)

Three reasonable reaction pathways for the deamidation of protonated AQ are shown in Scheme 1. The C-terminal oxygen can attack the glutamine side chain leading to 8, characterized by a glutaric anhydride ring; the amide bond nitrogen can attack the glutamine side chain resulting in 7, having a 5-membered γ-lactam ring; and NH_3_ can be lost from the N-terminus by concomitant nucleophilic attack by the C-terminal oxygen leading to the diketo-morpholine structure 9. Although structure 10 has a 7-membered ring, we have included it here as it was previously suggested as the NH_3_-loss product of protonated ValGln [35].

**Figure 3:**
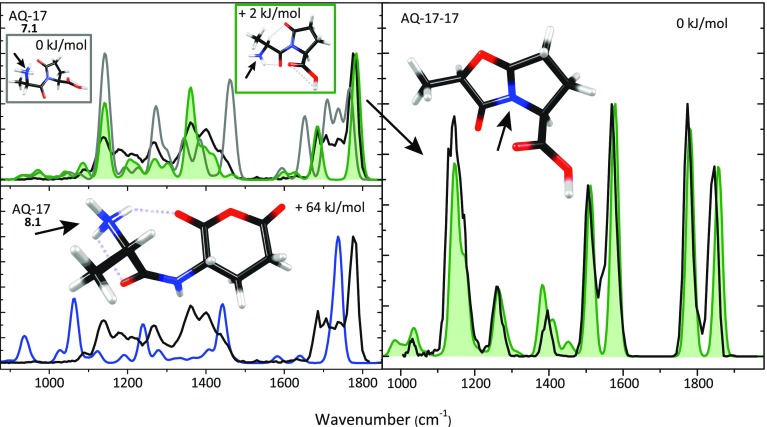
Experimental IRMPD spectrum of AQ-17 (*left panels*, *black*) compared with computed spectra for structures 7.1 (two conformers in *green and grey*) and 8.1 (*blue*). The *right panel* shows the experimental IRMPD spectrum of AQ – 17 – 17 (*black*) along with the spectrum predicted for a structure having expelled NH_3_ from the N-terminus of 7.1 with concomitant ring closure by nucleophilic attack of the succinimide oxygen onto the α-carbon of the Ala residue. Protonation sites are indicated with an *arrow*

Table 3 shows that the 5-membered lactam ring structure with the added proton at the N-terminus (7.1) has the lowest energy. Alternative structures are in this case substantially higher in energy, with the glutaric anhydride structure protonated at the N-terminus (8.1) being the next higher isomer at + 64 kJ/mol. Figure 3 shows the calculated absorption spectra of structures 7.1 (two nearly iso-energetic conformers in grey and green) and 8.1 (blue) compared with the experimental IRMPD spectrum (in black).

**Table 3:**
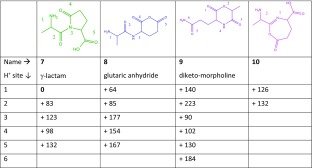
Deamidation of Protonated AlaGln (AQ).

Calculated relative free energies at 298 K for different possible isomers generated by NH_3_ loss, each having various possible protonation sites

Structure 8.1 would be formed if the deamidation of AQ followed a reaction mechanism analogous to that identified above for deamidation of protonated AN. However, as observed clearly in the bottom left panel of Fig. 3, the predicted IR spectrum for 8.1 does not match with the experimental spectrum. We conclude that AQ detaches NH_3_ by a different mechanism compared with its asparagine analogue. Furthermore, no match was found with computed spectra for the various protomers of 9 nor with those for the previously suggested structure 10, featuring a 7-membered ring. Supporting Information Figure S3 shows spectral comparisons with the computed spectra for 9.3, 10.1, and 10.2.

The top left panel of Fig. 3 shows the experimental spectrum of AQ – 17 compared with computed spectra for two conformers of the lowest energy protomer of the lactam ring containing product (7.1), having computed relative free energies within 2 kJ/mol. For the conformer shown in green, characterized by H-bonding between the protonated N-terminus and the amide carbonyl (rather than with the lactam carbonyl for the conformer shown in gray), the match between experimental and computed spectra is reasonable. The 1780 cm^–1^ band is assigned as being attributable to the two unresolved C=O bands in the lactam ring and in the carboxyl group, computed at 1781 and 1783 cm^–1^. The peak at 1680 cm^–1^ is due to the N-terminal ammonium group. We cannot exclude the presence of a minor fraction of the alternative conformer (grey) in the ion population, especially given the absorption observed around 1720 and 1460 cm^–1^. Further evidence for the assignment of the γ-lactam ring structure is provided by analysis of the structure of the MS^3^ fragment ion.

Activation of the isolated *m/z* 201 fragment induces the loss of a second NH_3_ molecule giving *m/z* 184 (AQ – 17 – 17), in line with observed MS^3^ behavior in [34]. Detachment of the N-terminal ammonium group from 7.1 with concomitant nucleophilic attack of the lactam oxygen onto the α-carbon of the Ala residue leads to a bicyclic structure shown in the right panel of Fig. 3. The calculated spectrum of this AQ – 17 – 17 fragment is shown in green and matches closely with the experimental spectrum. The bands at 1860 and 1780 cm^–1^ are due to the C=O stretch modes of the oxazolone and carboxyl moieties, respectively, whereas the band at 1580 cm^–1^ is due to stretching of the CN bond joining the two 5-membered rings. The bands around 1400–1500 cm^–1^ are due to several C-H bending modes in the molecule.

**Scheme 4:**
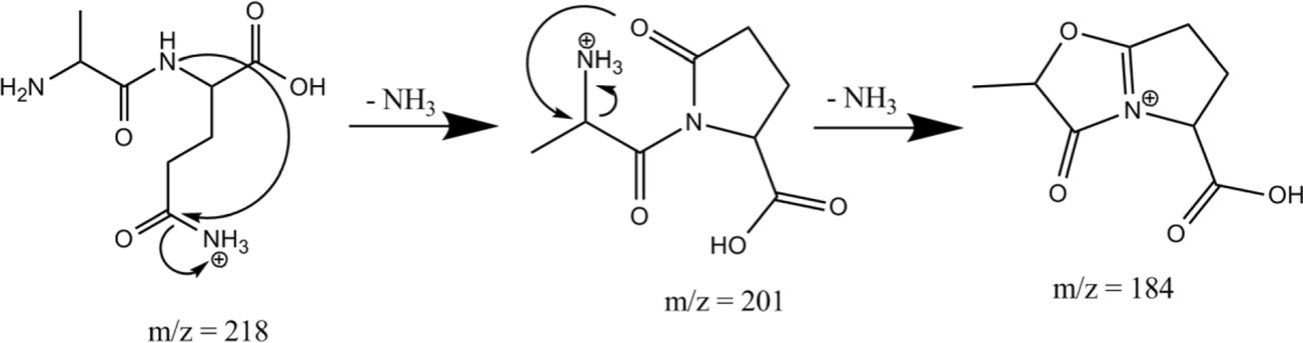
Reaction mechanism for the fragmentation of protonated AQ as suggested from the product ion structures identified from their IRMPD spectra

Identification of the bicyclic structure as the CID product of ammonia loss from AQ – 17 supports our above identification of the precursor AQ – 17 species as the lactam-containing ion 7.1. Scheme 4 summarizes the proposed reaction mechanism, which is based on the spectroscopically identified structures. This sequence of side chain and N-terminal ammonia loss is in agreement with what has been suggested by Harrison [34], although the structure of the MS^3^ fragment could not be determined in that study.

### Glutamine-Alanine (QA)

Of the dipeptides studied here, QA has the largest number of possible deamidation reaction mechanisms leading to 5- or 6-membered ring structures. Table 4 lists the six structures resulting from the suggested reaction mechanisms in the bottom row of Scheme 1. Nucleophilic attack by the N-terminal nitrogen onto the side chain and nucleophilic attack of the side chain nitrogen onto the α-carbon of the Gln residue lead to isomerically identical structures, although they can exist in two tautomeric forms indicated as 11 (enol) and 12 (keto) in Table 4. In its keto form, this structure is usually referred to as the pyroglutamyl product often suggested to result from NH_3_-loss from peptides with an N-terminal Gln residue [6–8, 34]. Depending on their site of protonation, the two tautomers may become equivalent, in particular, structures 11.1 and 12.1 are equivalent. The 6-membered δ-valerolactone derivative 13 is the result of a nucleophilic attack by the oxygen of the amide linkage onto the side chain. A nucleophilic attack of the amide bond nitrogen on the side chain leads to the glutarimide structure 14; this mechanism has been suggested for dehydration of peptides containing an N-terminal Glu residue [34]. Loss of NH_3_ can also occur from the N-terminus through attack of the side chain oxygen leading to 15 or through attack of the C-terminal oxygen leading to 16.

**Table 4:**
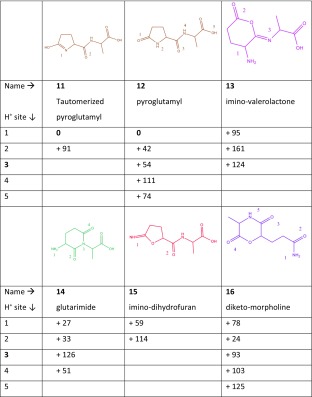
Deamidation of Protonated GlnAla (QA).

Calculated relative free energies at 298 K for different isomeric structures possibly resulting from NH_3_-loss, for different protonation sites for each of them. Note that 11 and 12 are tautomers of the same isomer; depending on the protonation site, the two structures may be equivalent (i.e., 11.1 is the same as 12.1)

**Figure 4:**
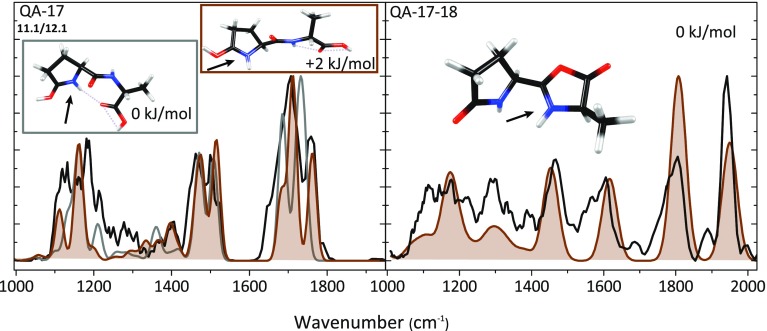
(*left*) Measured IRMPD spectrum (*black*) of QA – 17 compared with computed spectra (*grey and shaded brown*) for two nearly iso-energetic conformers of the lowest energy isomer 11.1 (see Table 4). (*right*) Measured spectrum for QA – 17 – 18 (*black*) along with the computed spectrum for the structure resulting from H_2_O loss from the C-terminus. These spectra suggest that product ions are formed on the reaction path indicated in *brown* in Scheme 1 and more detailed in Scheme 5

The left panel in Fig. 4 shows the experimental spectrum for the QA – 17 product ion along with computed spectra for two nearly iso-energetic conformers of the lowest energy isomer 11.1 in Table 4 (see Supporting Information Fig. S4 for a comparison with other structures listed in Table 4). The 0 kJ/mol conformation has a slightly more compact structure than the conformer computed at + 2 kJ/mol. The compact conformation is induced by an H-bond between the carboxylic carbonyl oxygen and the NH of the pyroglutamyl ring, while in the more extended conformer this O-atom forms an H-bond with the amide NH. The overall shape of the spectroscopic feature observed between 1600 and 1800 cm^–1^ suggests that the extended conformer, whose predicted spectrum is shown in brown in the left panel of Fig. 4, is the dominant contributor to the observed spectrum. On the other hand, the compact structure, the predicted spectrum of which is shown in gray, cannot be excluded and especially the feature observed near 1200 cm^–1^ suggests a significant contribution. For the extended structure of QA – 17, the band at 1760 cm^–1^ is due to the carboxyl C=O stretch, the band at 1710 cm^–1^ to C=O stretching of the peptide bond, and the band at 1680 cm^–1^ is due to CN stretching in the pyroglutamyl moiety. The bands around 1500 cm^–1^ are attributed to C-H bending.

**Scheme 5:**
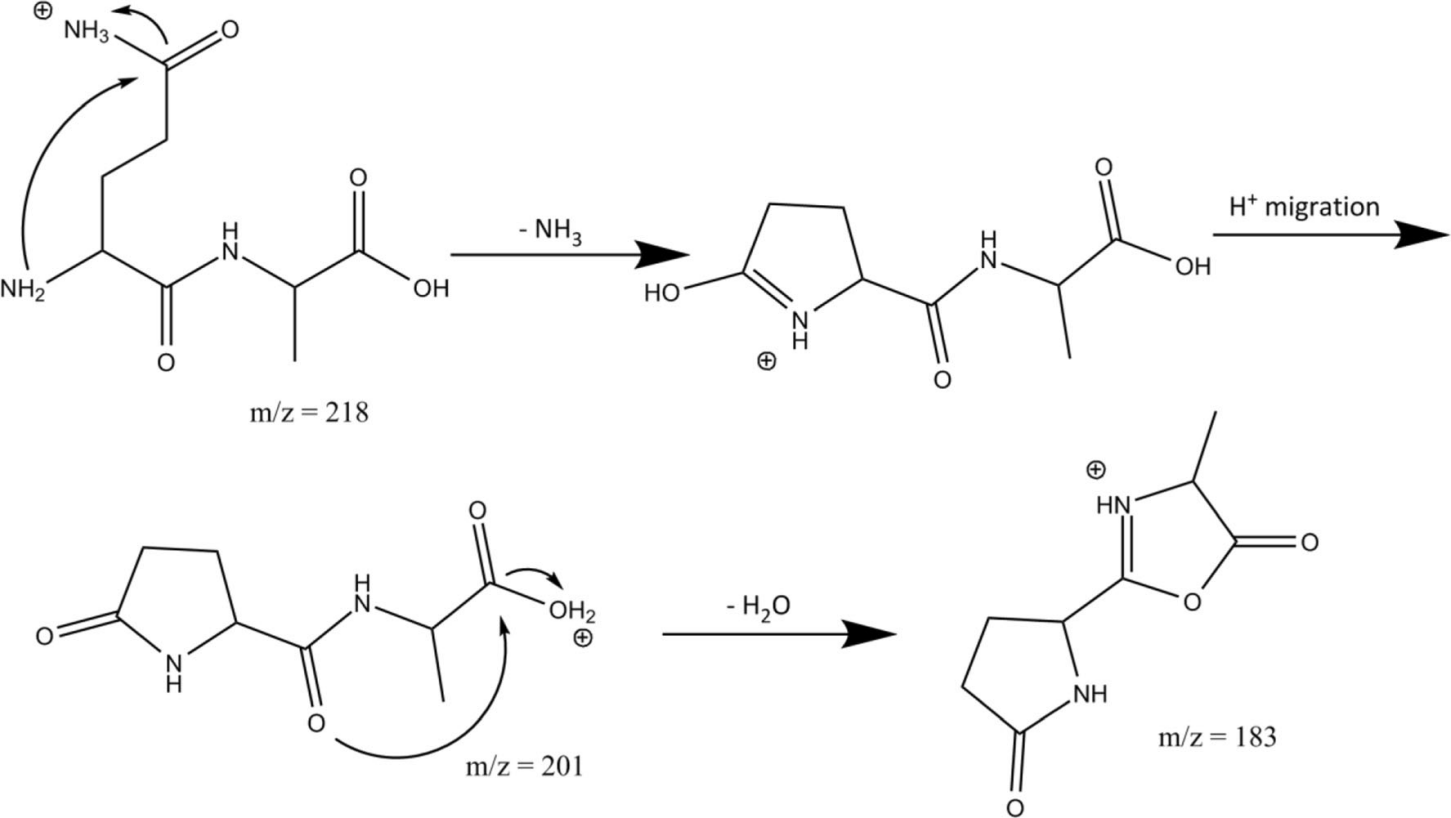
Suggested reaction mechanism for the sequential loss of NH_3_ and H_2_O from protonated QA as derived from the product ion structures identified from their IRMPD spectra

MS^3^ of QA – 17 did not provide QA – 17 – 17 as observed for AQ – 17. This is in line with the QA – 17 structure identified on the basis of the spectrum in the left panel of Fig. 4, from which it is not obvious how to expel another NH_3_. Instead, the main reaction channel observed involves H_2_O loss giving QA – 17 – 18. The structure of this MS^3^ fragment is also determined by IRMPD spectroscopy as shown in the right panel of Fig. 4. Water loss from the C-terminus is conceivable after H^+^-migration and nucleophilic attack by the amide carbonyl oxygen onto the C-terminal carboxyl carbon. This leads to the formation of an oxazolone ring protonated on the oxazolone nitrogen. Alternative protonation sites produce structures with relative free energies that are at least 20 kJ/mol higher. For this γ-lactam-oxazolone bicyclic structure of QA – 17 – 18, a spectrum is predicted with strong bands at 1950 cm^–1^ due to the oxazolone C=O stretch, at 1800 cm^–1^ due to the lactam carbonyl stretch, at 1610 cm^–1^ due to oxazolone CN stretching, and at 1450 cm^-1^ due to C-H bending in the molecule. This predicted spectrum matches the experimental spectrum for QA – 17 – 18 reasonably well so that we can summarize the breakdown of protonated QA as shown in Scheme 5. Note that for the NH_3_-loss in the first reaction step leading to the well-known pyroglutamyl ring, a nucleophilic attack from the N-terminus on the side chain is spectroscopically indistinguishable from a nucleophilic attack from the side chain to the N-terminus.

## Conclusion

**Figure 5:**
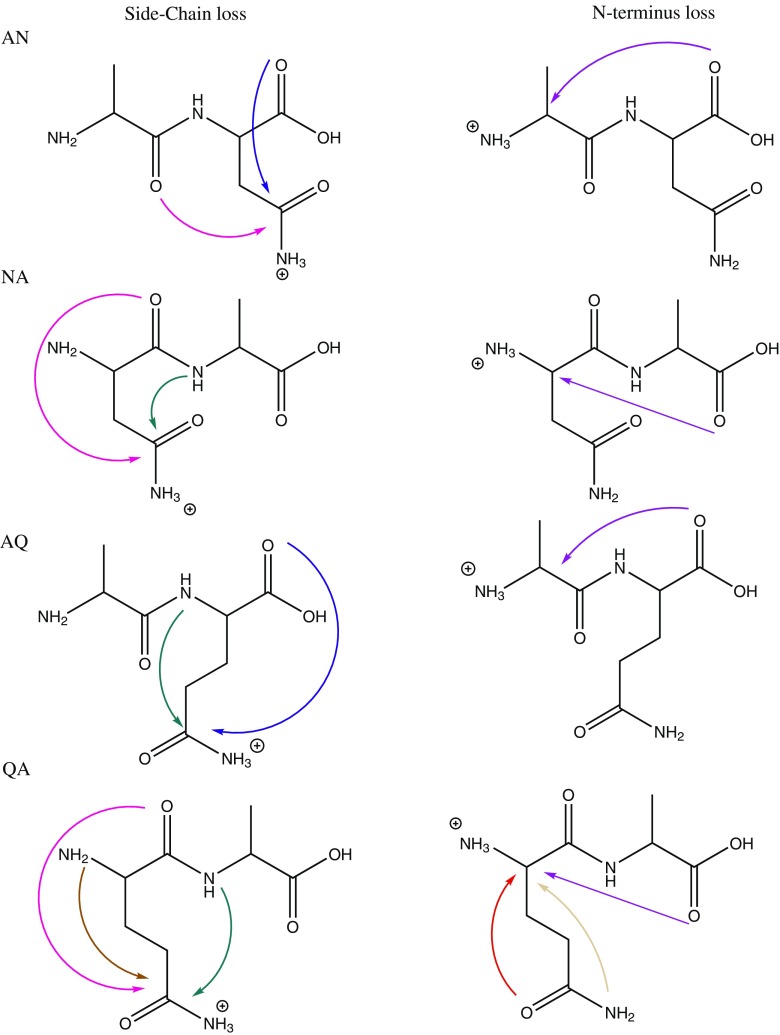
Molecular structure map of deamidation reactions in the Asn and Gln containing dipeptides based on the fragment ion structures established on the basis of their IR spectra

The sensitivity and versatility of a commercial ion trap mass spectrometer combined with a powerful tunable IR laser and quantum-chemical calculations provides ample opportunities to apply ion spectroscopy for deep molecular structure probing in MS^n^ reaction networks, from which reaction mechanisms can be qualitatively inferred. In this study, we showcase these possibilities by constructing a reliable and comprehensive molecular structure map of the deamidation reaction mechanisms occurring in protonated peptides containing Asn and Gln residues (see Fig. 5). These suggested mechanisms should form a useful starting point for computational modeling of the potential energy surfaces upon which the reactions proceed [47, 48].

Deamidation of protonated AN leads to a succinic anhydride structure, in contrast to what had been suggested for the deamidation of protonated VN on the basis of computational investigation alone [35]. This reaction is also different from that occurring in solution, where succinimide-like structures are formed [3–5]. The mechanism established here for the peptide with Asn in the C-terminal position is analogous to that found for deamidation of the protonated Asn amino acid [10, 33]. Deamidation of protonated NA proceeds along two bifurcating reaction pathways as evidenced by different spectral signatures observed in different IR dissociation channels. Nucleophilic attack of the peptidyl amide oxygen on the side chain generates a structure featuring a 5-membered lactone ring. Alternatively, a succinimide product ion is formed upon nucleophilic attack by the peptidyl amide nitrogen onto the side chain carbonyl carbon. These mechanisms are in accord with recently established pathways for deamidation of protonated NG [36].

For AQ, the sequential loss of two NH_3_ molecules is in agreement with earlier MS/MS studies [34], and here we add product ion structures and suggest reaction mechanisms. Elimination of NH_3_ from the side chain is induced by nucleophilic attack by the peptidyl amide nitrogen leading to a C-terminal pyroglutamic acid moiety. Subsequent elimination of NH_3_ from the N-terminus leads to a structure with two fused 5-membered rings. For deamidation of protonated QA, formation of a pyroglutamyl residue at the N-terminus is established, in line with common knowledge for peptides with an N-terminal Gln residue [3, 6–8].

One thing that all reaction mechanisms have in common is the fact that deamidation occurs in all four dipeptides from the side chain amide group, which is also the case for the Gln and Asn amino acids [10]. Moreover, all four dipeptides follow a reaction mechanism analogous to that in the respective amino acids, in the sense that the nucleophile driving the expulsion of an ammonia molecule is identical to the one in the amino acids. This is exemplified by deamidation of protonated AN and NA, induced by nucleophilic attack of the backbone carbonyl oxygen of the Asn residue. Deamidation product ions from protonated AQ and QA possess a pyroglutamate-like structure induced by nucleophilic attack by the backbone nitrogen of the Gln residue, analogous to the mechanism in the glutamine amino acid. Only for NA, a parallel mechanism unlike that in the amino acid has been uncovered, involving the backbone nitrogen atom as the nucleophile.

The different side chain length of Asn and Gln implies that if AQ followed the same mechanism as AN, and QA followed the same mechanism as NA, the higher energy 6-membered ring structures 8 and 13 would have formed for AQ and QA, respectively (see Tables 3 and 4). Instead, the backbone amide nitrogen acts as nucleophile leading to lower energy 5-membered ring structures. Similarly, if this reaction would occur for AN and NA, it would lead to unfavorable 4-membered ring structures. The difference in side chain length thus has decisive influence on the deamidation reaction mechanisms: for Asn-containing peptides, the nucleophile is the carbonyl O-atom at the C-terminal side of the residue, whereas for Gln-containing peptides, the nucleophile is the backbone N-atom on the N-terminal side of the residue.

## Electronic supplementary material

Below is the link to the electronic supplementary material.

ESM 1 (PDF 1.56 mb)
