# Supplementary material for: Deamidation Reactions of Asparagine- and Glutamine-Containing Dipeptides Investigated by Ion Spectroscopy
Source: J Am Soc Mass Spectrom. 2016 Sep 13;27(11):1855–69. doi: 10.1007/s13361-016-1462-5 (PMC5059420; doi:10.1007/s13361-016-1462-5)
Supplement: Supplementary file 1 — (PDF 1.56 mb) [file 13361_2016_1462_MOESM1_ESM.pdf]

Deamidation reactions of asparagine and glutamine containing dipeptides investigated by ion spectroscopy

Lisanne J.M. Kempkes[1], Jonathan Martens[1], Josipa Grzetic[1], Giel Berden[1], Jos Oomens[1,2,*]

[1]Radboud University, Institute for Molecules and Materials, FELIX laboratory, Toernooiveld 7c, 6525 ED Nijmegen, The Netherlands

[2]Van 't Hoff Institute for Molecular Sciences, University of Amsterdam, Science Park 904, 1098 XH Amsterdam, the Netherlands

Supporting Information

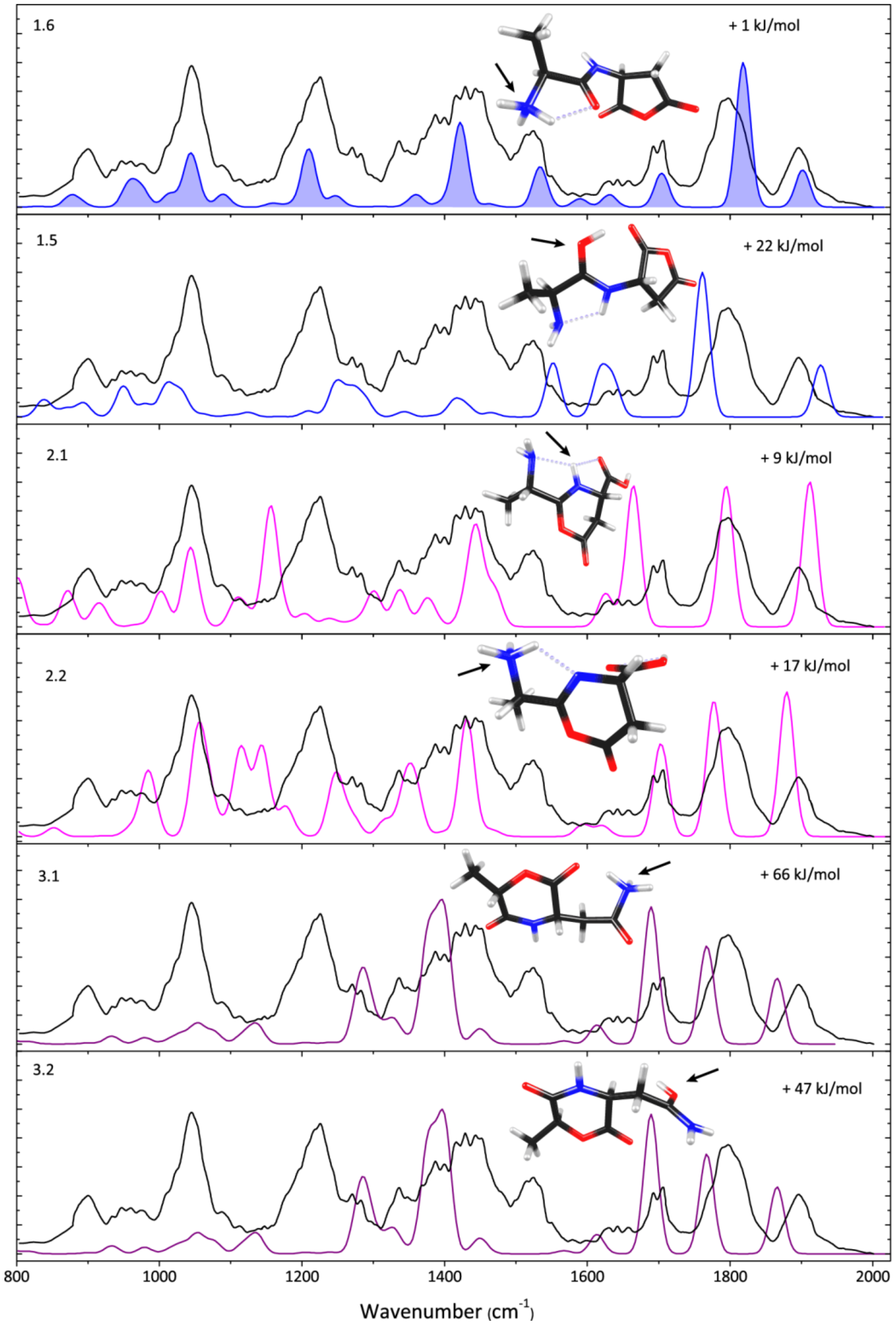

Figure S1. *IRMPD spectrum of AN – 17 compared with the assigned structure calculation (shaded blue spectrum) and alternative structures from Table 1 having relative energies lower than 50 kJ/mol.*

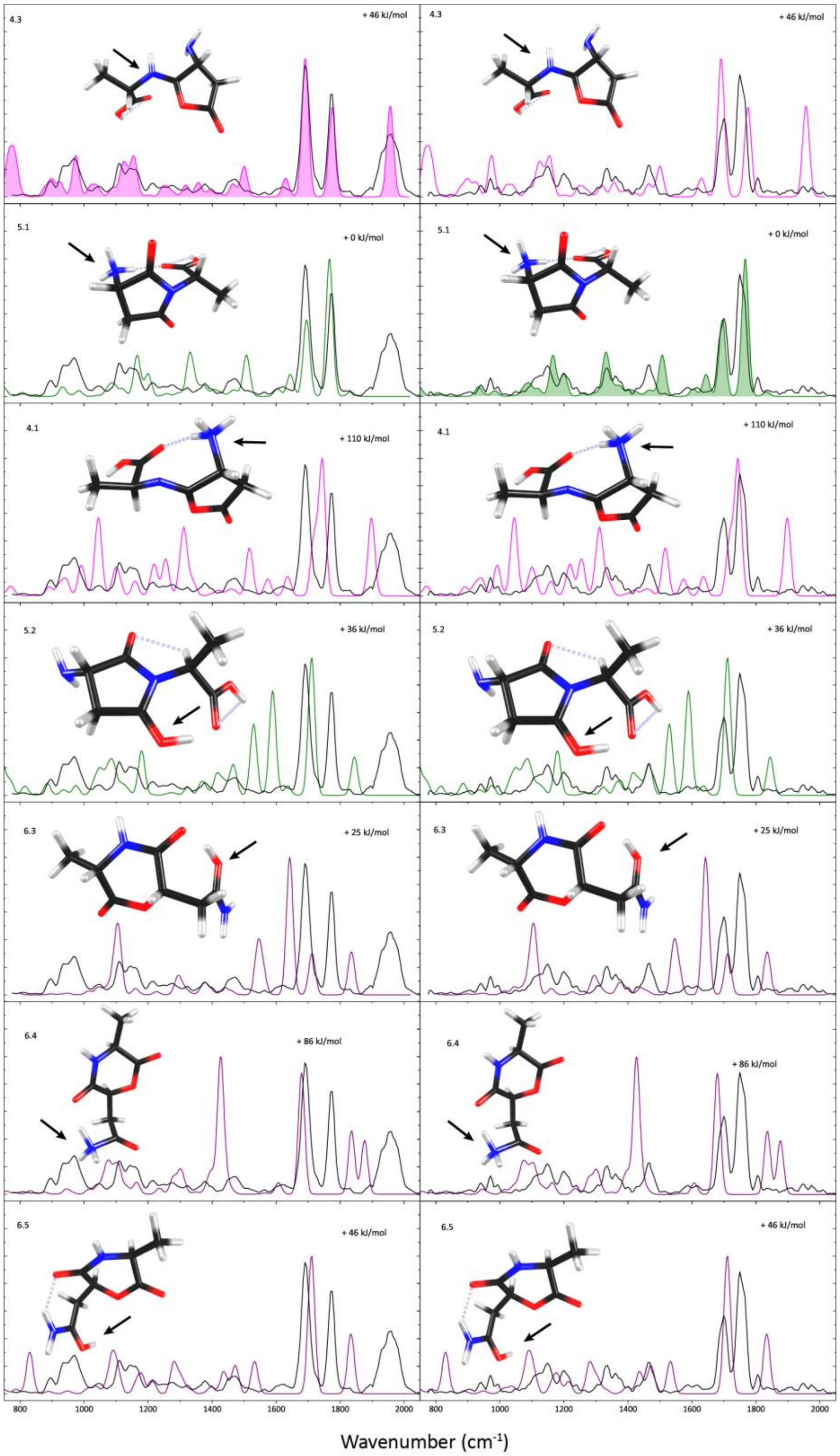

Figure S2. *IRMPD spectra of NA – 17 compared with the computed spectra for alternative structures from Table 2.*

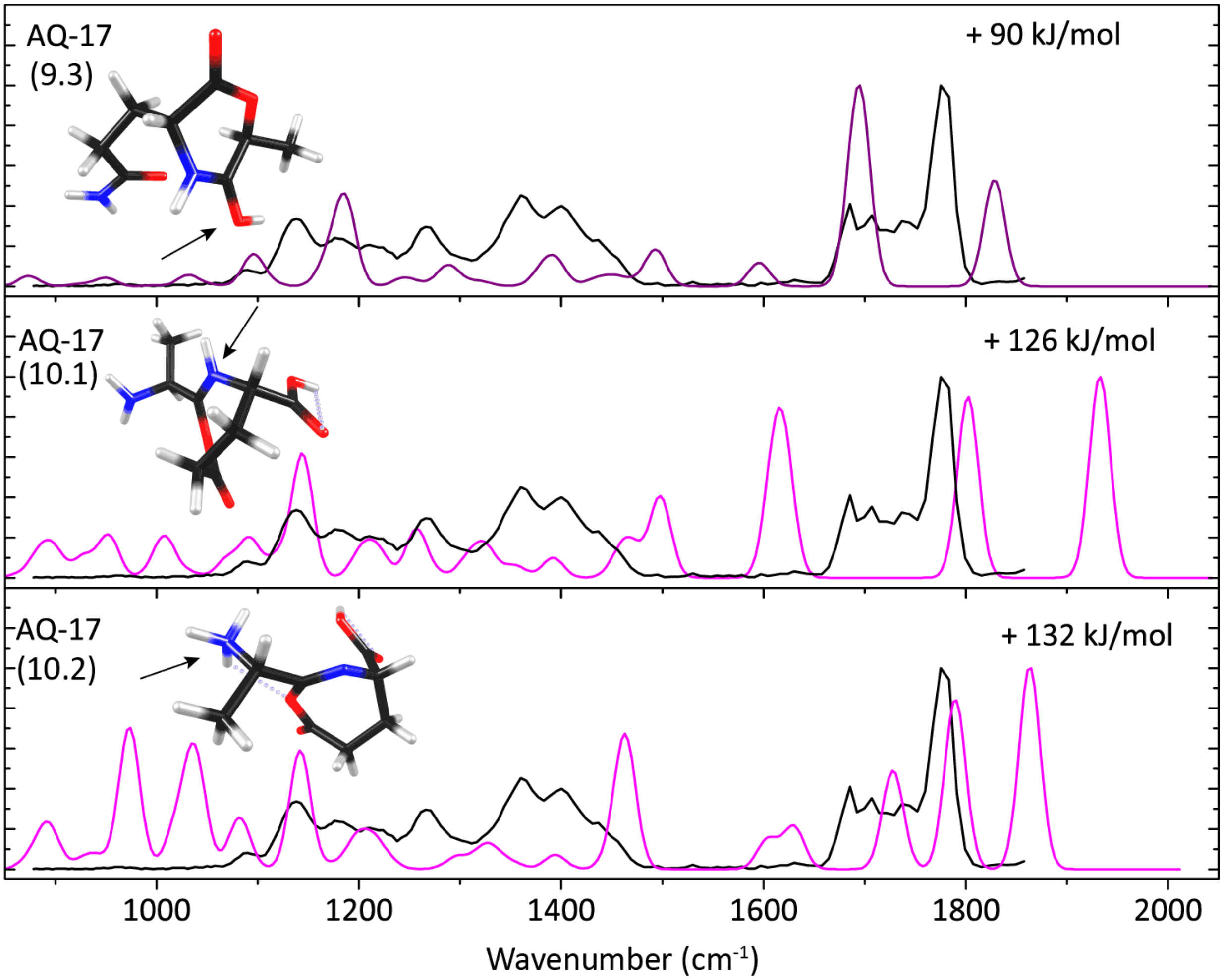

Figure S3. *IRMPD spectrum of AQ – 17 compared with the predicted spectrum of structure* ***9.3*** *(see Table 3) and with those of the previously suggested 7-membered ring structures* ***10.1*** *and* ***10.2****.*

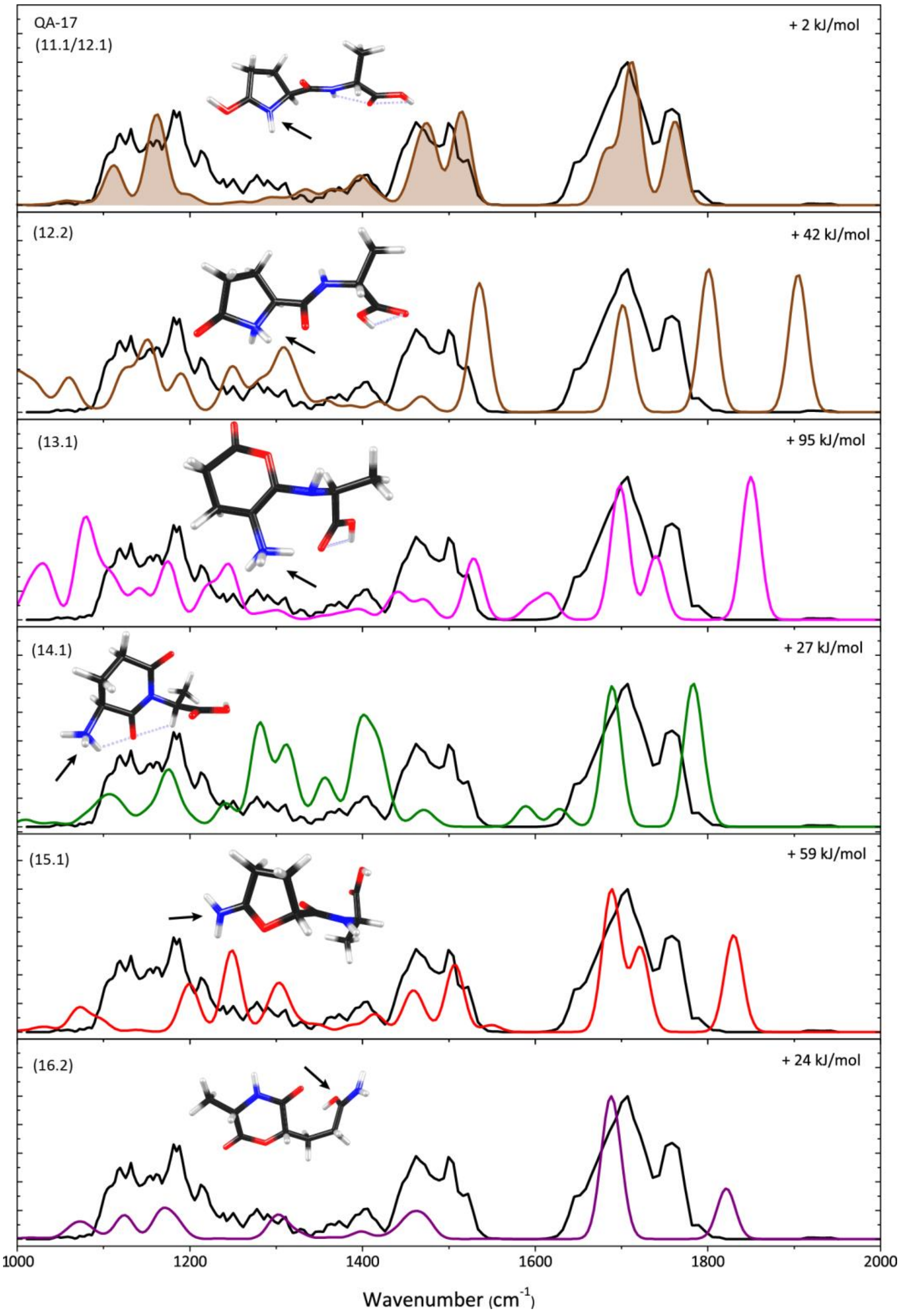

Figure S4. *IRMPD spectrum of QA – 17 compared with the computed spectra for the assigned structure (shaded brown spectrum) and some alternative structures from Table 4.*

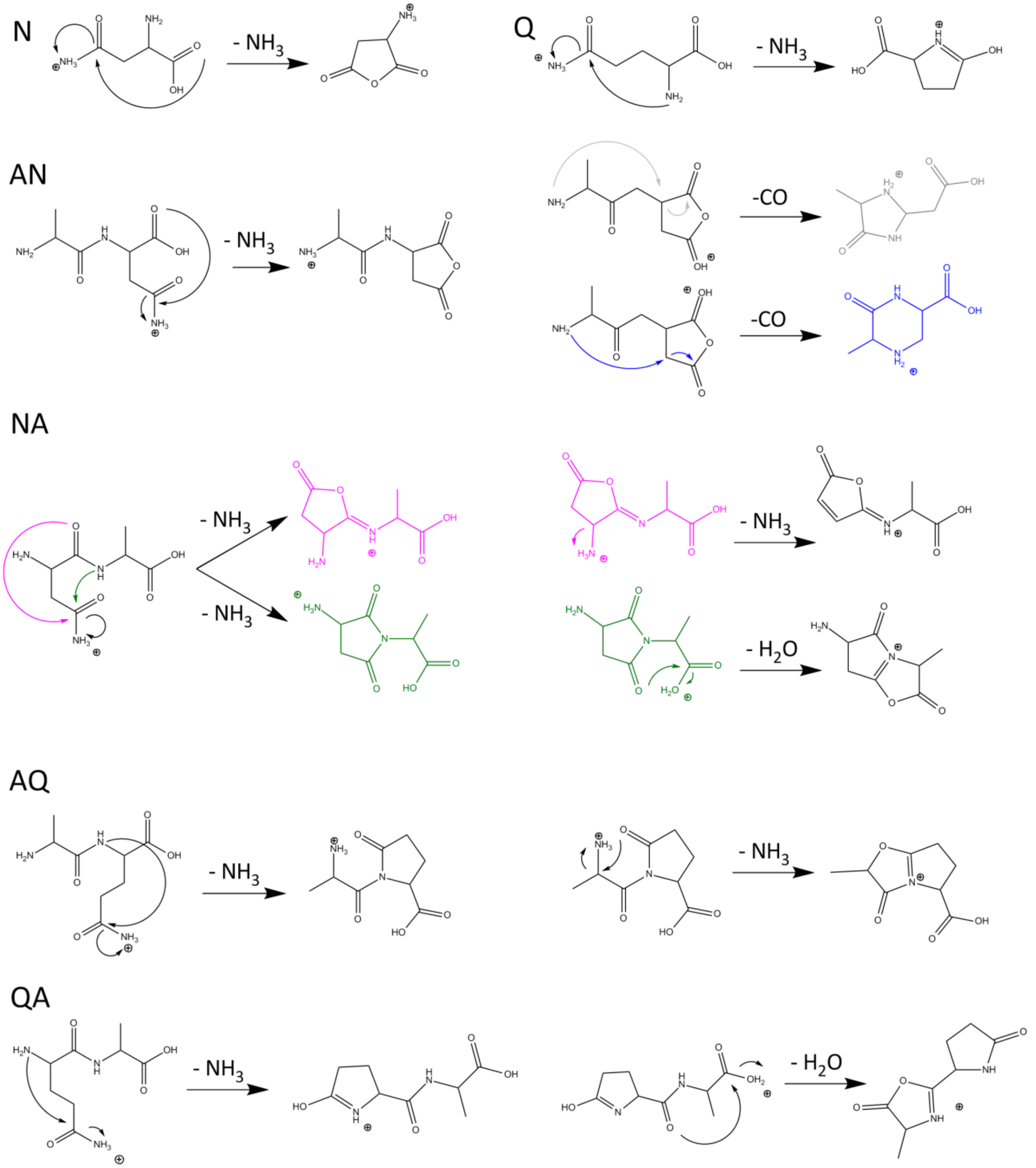

Scheme S1: *Overview of identified MS^2 and MS^3 reaction pathways for protonated N and Q compared with those of the dipeptides AN, NA, AQ and QA.*
